# The Relationship between Pinguecula and Diabetes Mellitus: A Comparative Cross-Sectional Study

**DOI:** 10.1155/2023/9060495

**Published:** 2023-02-28

**Authors:** Hisham M. Jammal, Mohammed Abu-Ameerh, Jamila G. Hiasat, Sara Issa, Muawyah Al Bdour

**Affiliations:** ^1^Department of Ophthalmology, Faculty of Medicine, Jordan University of Science and Technology, Irbid, Jordan; ^2^Department of Ophthalmology, Jordan University Hospital, The University of Jordan, Amman, Jordan; ^3^Division of Pediatric Ophthalmology and Strabismus, UPMC Children's Hospital of Pittsburgh, PA, USA; ^4^Department of Ophthalmology, University of Pittsburgh School of Medicine, PA, USA; ^5^Department of Ophthalmology, King's College Hospital, London, UK

## Abstract

**Purpose:**

To assess the relationship between diabetes mellitus (DM) and the presence of pinguecula and to identify other risk factors associated with pinguecula in patients attending the eye clinic at two tertiary university hospitals in Jordan.

**Methods:**

This was a comparative cross-sectional hospital-based study of 241 consecutive patients (122 patients with DM and 119 patients with no diabetes). All patients underwent complete ophthalmic examination, and data were collected regarding age, sex, occupational activity, presence and grade of pinguecula, glycosylated hemoglobin (HbA1c), and presence of diabetic retinopathy.

**Results:**

The mean (standard deviation, SD) ages of the DM and non-DM groups were 59.5 (10.8) years and 59.0 (11.6) years (*p*-value = 0.729), respectively. There was no significant difference in the prevalence of pinguecula between the diabetic and nondiabetic groups (66.4% vs. 66.5%, *p* = 0.998). Multivariate logistic regression analysis revealed that only outdoor occupational activity (OR = 5.16, 95% CI: 1.98–13.44, *p* = 0.001) was associated with increased prevalence of pinguecula. DM was not significantly associated with pinguecula (OR = 0.96, 95% confidence interval (CI): 0.55–1.67, *p* = 0.873). Neither age nor sex were significantly associated with pinguecula (*p*-value = 0.808, *p*-value = 0.390), respectively.

**Conclusion:**

DM was not significantly associated with the development of pinguecula in this Jordanian population. The prevalence of pinguecula was significantly associated with an outdoor occupational activity.

## 1. Introduction

Pinguecula is a common benign and degenerative condition of the bulbar conjunctiva that is characterized by an elevated yellowish lesion on either side of the cornea [1]. Histologically, the lesion demonstrates elastotic changes in collagen fibers resulting in the formation of basophilic subepithelial tissue [1]. The exact pathogenesis is still incompletely understood; most studies associate pinguecula with increasing age and sunlight exposure [2–6]. Other less clear associations include alcohol intake [4], dry eye syndrome [7], and contact lens wear [8, 9].

To further explore the molecular basis of the relation between ultraviolet exposure and pinguecula, Kaji et al. [10] detected significant amounts of advanced glycation end-products (AGEs) in pinguecula surgical specimens compared to specimens of conjunctiva without pinguecula; they attributed this to local ultraviolet (UV) irradiation and decreased antioxidant activities. Diabetes mellitus (DM) exerts systemic complications by producing AGEs [11] and may thus be a potential factor in the development of pinguecula. Some corneal manifestations of DM may be attributed to the accumulation of AGEs in corneal basement membranes [12]. Mimura et al. [13] suggested that diabetes may be associated with the development of pinguecula. To the best of our knowledge, no further studies were conducted to describe this association. This study aimed to assess the relationship between DM and pinguecula in patients attending the eye clinic at two tertiary university hospitals in Jordan.

## 2. Materials and Methods

### 2.1. Study Design

A comparative cross-sectional hospital-based study was conducted in a group of 122 consecutive patients with DM and a group of 119 patients with no DM, age-matched by group, who attended the ophthalmology clinic at two tertiary teaching hospitals in Jordan (Jordan University Hospital and King Abdullah University Hospital). All patients were Jordanians of Arab ethnicity.

In the DM group, DM was diagnosed if patients were currently treated with oral hypoglycemic agents, with or without insulin, and if they fulfilled the American Diabetic Association (ADA) Guidelines diagnostic criteria [14]. In the non-DM group, all patients had glycosylated hemoglobin (HbA1c) <5.8%. Those with a history of ocular surgery involving the ocular surface or nasolacrimal apparatus, eyelid abnormalities (e.g., entropion and trichiasis), acute conjunctivitis, recent use of topical ophthalmic medication (e.g., steroids), and history of wearing contact lenses were excluded.

The study was approved by the Ethics Committee for Medical Research at the Jordan University Hospital and the University of Jordan. It adhered to the tenets of the Declaration of Helsinki. Informed consent for participation was obtained from all participants.

### 2.2. Data Collection

Demographic and clinical data including age, sex, occupational activity, and presence and duration of DM were collected using a structured data collection form. The outdoor occupational activity was considered if ≥4 hours of work was outdoors. All patients underwent a complete ophthalmic examination including visual acuity testing, slit lamp examination, and dilated fundus examination. Data on the presence, severity, laterality, and location of pinguecula in affected eyes, and the presence and severity of diabetic retinopathy were recorded.

Pinguecula was diagnosed when present in either eye. The severity of pinguecula was graded according to a scheme based on slit lamp examination findings (Table 1) [13].

### 2.3. Statistical Analysis

Data were analyzed using Statistical Package for Social Sciences (SPSS) version 23 (SPSS Inc., Chicago, IL, USA). Frequencies and percentages were calculated for categorical data and Pearson's chi-square test was used to assess the association between the presence of pinguecula and DM and other categorical variables. The independent samples *t*-test was used to compare means between the two groups. The association between DM (independent variable) and pinguecula (dependent variable) was analyzed using binary logistic regression to adjust for possible factors. A *p*-value of <0.05 was considered statistically significant.

## 3. Results

### 3.1. Baseline Characteristics

There were 122 patients (50.6%) with DM and 119 (49.4%) without diabetes. The baseline characteristics of the two study groups are shown in Table 2. The mean (SD) ages of the DM and non-DM groups were 59.5 (10.8) years and 59.0 (11.6) years, which were not significantly different (*p*-value = 0.729). Differences in sex and outdoor/indoor occupation between the two groups were not significant (*p*-value = 0.219 and *p*-value = 0.423, respectively). The mean (SD) duration of DM in the DM group was 12.3 (7.1) years (range, 1–32 years).

### 3.2. Prevalence of Pinguecula

The prevalence of pinguecula among patients with and without DM were 66.4% and 65.5%, respectively and were not significantly different (*p*-value = 0.998). In the DM group, there was no significant association between the presence of pinguecula and the stage of diabetic retinopathy (*p*=0.285), the duration of DM (*p*=0.647), or the level of HbA1c (*p*=0.160).

Among patients with a pinguecula, no association between the presence of diabetes and severity or laterality (*p*-value = 0.094 and *p*-value = 0.313, respectively) of the pinguecula was found. Table 2 summarizes the differences between patients with DM and patients with no DM.

### 3.3. The Multivariate Analysis of the Association between DM and Pinguecula

In multivariate logistic regression, the association between DM and pinguecula was tested after adjusting for sex, occupational activity, and age (Table 3). After adjusting for important variables in the model, diabetes was not significantly associated with pinguecula (odds ratio (OR) = 0.96, 95% confidence interval (CI): 0.55–1.67, *p*=0.873). The only variable significantly associated with pinguecula was outdoor occupational activity (OR = 5.16, 95% CI: 1.98–13.44, *p*=0.001).

## 4. Discussion

Few studies assessed the prevalence and risk factors for pinguecula and found a wide range of 11.3–75.6% prevalence rates around the world, reflecting the heterogeneity in the study designs (population versus hospital-based), age groups, population characteristics (e.g., social, environmental, ethnic, and geographic factors) related to UV radiation levels [3, 4, 15–20].

None of the population-based studies on the prevalence of pinguecula that included DM in the assessment found a relationship between the two [3, 4, 15]. However, results from the hospital-based study by Mimura et al. [13] suggested that DM may be associated with the development of pinguecula, possibly due to deposition of AGEs, diabetic microvascular damage of conjunctival blood vessels, and diabetes-related eye dryness. We could not find a significant association between diabetes and the presence of pinguecula, nor between the severity of pinguecula and diabetes. The lack of association between pinguecula and duration of DM, HbA1c levels, and the stage of diabetic retinopathy among patients with DM in the current study may further support the lack of association between DM and pinguecula. However, the effect of ethnic and genetic differences between the two populations should not be ignored, as racial and ethnic differences in diabetic complications and comorbidities do exist globally [21].

Most of the literature on the prevalence of pinguecula reports that pinguecula is significantly associated with age and outdoor occupational activity, both of which may indicate chronic UV light exposure. Data from the present study revealed a 66% prevalence of pinguecula among all study participants. This rate approximates those in Australia (69.5%) [20] and in two studies from Iran (56.2% and 61.0% in 40–59 years and 40–64 years age groups, respectively) [16, 19]. Interestingly, Norn [22] reported a much higher prevalence rate of 90% in the Red Sea territory of Jordan among a sample of 127 subjects, aged ≥10 years. The included subjects resided in the Red Sea port of Aqaba in Jordan, known to have a higher solar UV index than the rest of the country, and nearly half of the study subjects were soldiers, who were more likely to spend time outdoors. In addition, Norn [22] found that the percentage of pinguecula increased within the 10-year age groups of 10, 20, 30, and 40 years but leveled out after 40 years, which is similar to the roughly constant prevalence rates over the 10-year age groups above 40 years found in our study. To explain this stability, it is possible that exposure to UV radiation in sunlight initiates the formation of pinguecula at younger ages which increases in prevalence over time. Then, at older ages, subjects who did not develop pinguecula earlier may continue to remain unaffected, as they become less exposed to sunlight owing to retirement, sedentary lifestyle, or preference for indoor occupations. A high level of UV exposure in childhood is a risk factor for high rates of UV-related ocular disease in adulthood, according to Seki et al. [23], who reported a pinguecula prevalence rate of 29.1% among 227 children living in rural Tanzania.

In the current study, outdoor occupational activity was the only significant independent factor associated with pinguecula. This is consistent with findings from other studies [3, 4, 15, 19, 22]. The similar rates of pinguecula observed in countries located at different latitudes with different UV radiation levels indicate that environmental, ethnic, and genetic factors may interact with or modify the mechanism by which UV exposure contributes to the development of pinguecula.

In the present study, sex was not found to be significantly related to pinguecula after adjusting for other factors, contrary to most studies that found an association between the male sex and pinguecula [4, 16, 17, 19, 20]; we attributed this to how males tend to be more involved in outdoor occupations than females.

Some study limitations should be acknowledged. This was a hospital-based and cross-sectional study, which may suffer from selection bias. A longitudinal and cohort study design may help to better describe the association between DM and pinguecula. In addition, the use of sunglasses as protective measures against UV radiation was not evaluated.

In summary, no relationship between DM and pinguecula was found among Jordanians. The presence of pinguecula was associated with an outdoor occupational activity.

## Figures and Tables

**Table 1:** Pinguecula grading system.

Grade	Features
0	No pinguecula
1	Mild or moderate: yellowish white and flat or slightly elevated lesions with a maximum diameter <5 mm
2	Severe: highly vascularized and elevated lesions or large lesions ≥5 mm in diameter

**Table 2:** Patients' characteristics and variables in diabetic vs. nondiabetic groups.

Variables	DM (%)	Non-DM (%)	*P*-value
Age (years)			
40–49 years	30 (24.6)	30 (25.2)	0.998
50–59 years	32 (26.2)	30 (25.2)	
60–69	32 (26.2)	31 (26.1)	
≥70	28 (23.5)	28 (23.5)	
Sex			
Male	65 (53.3)	53 (44.5)	0.219
Female	57 (46.7)	66 (55.5)	
Occupational activity			
Indoor	90 (73.8)	94 (79.0)	0.423
Outdoor	32 (26.2)	25 (21.0)	
Presence of pinguecula	81 (66.4)	78 (65.5)	0.998
Grade of pinguecula			
Grade 0	41 (33.6)	41 (34.5)	0.178
Grade 1	55 (45.1)	63 (52.9)	
Grade 2	26 (21.3)	15 (12.6)	

DM: diabetes mellitus.

**Table 3:** Multivariate analysis of factors associated with pinguecula.

Variable	OR	95% C.I		*p*-value
		Lower	Upper	
DM	0.96	0.55	1.67	0.873
Sex (male versus female)	1.30	0.71	2.39	0.390
Occupational activity (outdoor versus indoor)	5.16	1.98	13.44	0.001
Age	1.00	0.97	1.02	0.808

OR: odds ratio; CI: confidence interval; DM: diabetes mellitus.

## Data Availability

The datasets used and/or analyzed during the current study are available from the corresponding author upon reasonable request.
